# The Scientific Impact Derived From the Disciplinary Profiles

**DOI:** 10.3389/frma.2020.569268

**Published:** 2020-10-16

**Authors:** Jüri Allik, Kalmer Lauk, Anu Realo

**Affiliations:** ^1^Institute of Psychology, University of Tartu, Tartu, Estonia; ^2^Grant Office, University of Tartu, Tartu, Estonia; ^3^Department of Psychology, University of Warwick, Coventry, United Kingdom

**Keywords:** disciplinary profiles, scientific impact, *Essential Science Indicators*, multidimensional scaling, bibliometrics

## Abstract

The disciplinary profiles of the mean citation rates across 22 research areas were analyzed for 107 countries/territories that published at least 3,000 papers that exceeded the entrance thresholds for the *Essential Science Indicators* (*ESI*; Clarivate Analytics) during the period from January 1, 2009 to December 31, 2019. The matrix of pairwise differences between any two profiles was analyzed with a non-metric multidimensional scaling (MDS) algorithm, which recovered a two-dimensional geometric space describing these differences. These two dimensions, Dim1 and Dim2, described 5,671 pairwise differences between countries' disciplinary profiles with a sufficient accuracy (stress = 0.098). A significant correlation (*r* = 0.81, *N* = 107, *p* < 0.0001) was found between Dim1 and the *Indicator of a Nation's Scientific Impact* (*INSI*), which was computed as a composite of the average and the top citation rates. The scientific impact ranking of countries derived from the pairwise differences between disciplinary profiles seems to be more accurate and realistic compared with more traditional citation indices.

## The Scientific Impact Derived From the Disciplinary Profiles

Although not perfect, the number of times a scientific paper has been cited since its publication is an objective and easy-to-determine indicator of its scientific impact, which was forecasted long before counting citations became practically feasible (Garfield, 1955). After an expected link between scientific and economic wealth was established—countries whose scientists tend to publish highly cited science papers had also higher level GDP per capita—the mean citation rate acquired a status of the most reliable measure of the scientific quality of nations (May, 1997; Rousseau and Rousseau, 1998; King, 2004; Harzing and Giroud, 2014; Prathap, 2017). However, it was noticed that some countries, for example, Sweden and Finland, seem to have lower mean citation rates than some other countries with a comparable level of scientific development such as Switzerland and the Netherlands (Karlsson and Persson, 2012; Öquist and Benner, 2015). It was also observed that Scandinavian papers published with international co-authorship produced a higher citation rate than purely domestic papers (Glänzel, 2000). It was also noticed that there was a gap between national mean citation rates and the proportion of highly cited papers that countries' scientists were publishing, which could be considered as an index of complaisance showing satisfaction with a relatively modest scientific ambitions (Allik, 2013; Lauk and Allik, 2018).

These and other similar problems caused a shift in the bibliometric research from impact scores based on average values of citations toward the use of indicators that reflect the top of the citation distribution, such as the number of papers reaching the highest rank of citations (van Leeuwen et al., 2003). In accordance with this general trend, a composite index—the *High Quality Science Index* (HQSI; Allik, 2013; Allik et al., 2020a)—was proposed characterizing nations by combining the mean citation rate per paper with the percentage of the papers that have reached the top 1% level of citations in a given research area and an age cohort of published papers. Although the average values of citations and the top of the citation distribution are highly correlated, typically *r* = 0.80 or higher (Allik, 2013), combining these two indicators into a composite index allowed to compensate some minor discrepancies between the two indicators.

Despite these improvements, the rankings of countries based on their citation frequencies are still often counterintuitive, seemingly at least. For example, very few experts would have expected that Panama will become a leading country whose scientists are publishing papers with the highest citation rate in the whole world (Monge-Najera and Ho, 2015; Confraria et al., 2017; Erfanmanesh et al., 2017; Allik et al., 2020a). One possible reason for such implausible rankings is that the selected top layer of papers is not representative of the total scientific production of a given nation (Allik et al., 2020a). When the *Essential Science Indicators* (*ESI*; Clarivate Analytics) database was designed, the whole science (except humanities) was decided to divide into 22 research areas with a quite different publication and citation rates. However, counting minimally required number of citations to enter the *ESI* in one of the research fields created a situation where it may be more advantageous to avoid entering *ESI* in relatively weak research areas that could decrease the country's average citation rate. As was shown by Allik and colleagues (2020a), leaving weaker publications out of counting may artificially increase the mean citation rate of that nation (Allik et al., 2020a). To deal with this problem, a new indicator—the *Indicator of a Nation's Scientific Impact* (*INSI*)—was proposed, wherein, in addition to the average and the top citation rates, the number of research areas in which each country/territory had succeeded to enter the *ESI* was also taken into account (Allik et al., 2020b). This modification made the scientific impact ranking of countries/territories more plausible, unfortunately not entirely. For example, the Republic of Georgia, which had the 5th highest mean citation rate, was shifted five positions down in the ranking because of the failure to exceed the *ESI* entrance threshold in 11 out of 22 scientific fields. However, Panama—also failing in 11 areas—dropped only two positions in the ranking and remained nevertheless ahead of the Netherlands, Denmark, and the United Kingdom not to mention USA, Canada, and Germany.

It was noticed that characteristics of the disciplinary structure may also be a factor that affects the competitive advantages of national sciences (Yang et al., 2012; Bongioanni et al., 2014, 2015; Cimini et al., 2014; Harzing and Giroud, 2014; Radosevic and Yoruk, 2014; Albarran et al., 2015; Lorca and de Andrés, 2019; Pinto and Teixeira, 2020). For example, it has been argued that this archaic disciplinary structure is one of the reasons why Russia and other former communist countries are still lagging behind Western nations (Kozlowski et al., 1999; Markusova et al., 2009; Adams and King, 2010; Guskov et al., 2016; Jurajda et al., 2017; Tregubova et al., 2017; Shashnov and Kotsemir, 2018). In a comprehensive study of how disciplinary structure is related to the competitive advantage in science of different nations, Harzing and Giroud (2014) showed that countries that demonstrated the fastest increase in their scientific productivity during the periods 1994–2004 and 2002–2012 remained relatively stable in their fairly well-balanced disciplinary structures. They also identified different groups of countries with distinct patterns of specialization. For example, one group of countries with a highly developed knowledge infrastructure had an emphasis on social sciences. Another group of countries had a rather balanced research profile with some slight advantage in physical sciences. Yet another group of countries mainly comprised Asian countries with a competitive advantage in engineering sciences (Harzing and Giroud, 2014). Although this study shed light on slightly different routes toward scientific excellence, it is still unclear whether there are truly separate routes or only one general highway, which guarantees advancement in the world ranking.

One of the problems with existing research on examining the scientific disciplinary profiles is that previous studies typically involved a relatively small number of nations. For example, the study by Harzing and Giroud (2014) analyzed disciplinary profiles of 34 countries across 21 disciplines while Almeida et al. (2009) examined disciplinary profiles of 26 European countries. Another study analyzed 27 European countries across 27 disciplines over the period from 1996 to 2011 (Bongioanni et al., 2014). Thelwall and Levitt (2018) analyzed the relative citation impact for 2.6 million articles from 26 fields in the 25 countries published from 1996 to 2015. Pinto and Teixeira (2020) examined disciplinary profiles of 65 countries over a broad period of time (1980–2016). There were several studies analyzing 16 G7 and BRICS countries (Yang et al., 2012; Shashnov and Kotsemir, 2018; Yue et al., 2018). Li (2017) explored disciplinary profiles of 45 countries, which is still a relatively small fraction of nations capable for a substantial scientific contribution. Estimating that there are about 100 nations with sufficiently advanced sciences, a need for more inclusive studies is obvious.

### The Aim of the Present Study

To advance the existing research, the aim of the present study is to examine the disciplinary profiles of the mean citation rates for 107 countries or territories whose scientists made substantial contributions to the world's essential science. In accordance with a recommendation to use indicators reflecting the top of the citation distribution (van Leeuwen et al., 2003), we used publications that were selected by the *ESI* based on their top citation rates. For each country/territory that had exceeded the entrance thresholds, their disciplinary profiles were formed based on their mean citation rates across 22 broad disciplines that *ESI* uses to monitor publication and citation performance.

When comparing disciplinary profiles of any two countries, we can judge how similar or dissimilar disciplinary strengths or weaknesses of these two countries are. From pairwise (dis)similarities between any two disciplinary profiles, it is possible to construct a matrix of distances between all countries/territories. By applying a multidimensional scaling (MDS) algorithm to this matrix, we may hope to recover from it a geometric space of low dimensionality, which could represent these data, as they are points in this geometric space (Borg and Groenen, 2005). If axes of this geometric space have a meaningful interpretation, then we may have a novel way for the construction of a new index characterizing the scientific impact of nations, which would not base on the average or the top values of the citation distribution alone.

## Methods

Data were retrieved from the latest available update of the *ESI* (*Clarivate Analytics*, updated on March 12, 2020; https://clarivate.com/products/essential-science-indicators/) that covered an 11-year period from January 1, 2009, until December 31, 2019. This update contained over 16 million *Web of Science* (*WoS*) documents, which were cited over 221 million times with an average frequency of 13.5 times per document.

In order to be included in the *ESI*, journals, papers, institutions, and authors need to exceed the minimum number of citations obtained by ranking journals, researchers, and papers in a respective research field in descending order by citation count and then selecting the top fraction or percentage of papers. For the authors and institutions, the threshold is set for the top 1% and the top 50% is established for countries and journals in an 11-year period. The main purpose of dividing into the fields is to balance publication and citation frequencies in different research areas. The *ESI* entrance thresholds were quite different for the research areas. For example, in the field of clinical medicine, 16,012 citations were needed for a country/territory in order to pass the *ESI* threshold whereas the respective figures in the fields of mathematics and economics & business were 494 and 321.

Among 149 countries/territories that passed the *ESI* threshold at least in one research field were several that published a small number of papers. For example, researchers from the Seychelles, Bermuda, and Vatican published 421, 404, and 257 papers, respectively, which were able to surpass the disciplinary entrance thresholds during the last 11 years. To include countries with a sufficient number of papers, we analyzed only countries that published more than 3,000 papers during the 11-year period. This entrance threshold was slightly lowered compared with the previous studies where it was 4,000 (Allik, 2013; Lauk and Allik, 2018; Allik et al., 2020b) to include a maximally large number of countries/territories making substantial contribution to the world science. Applying this criterion, 107 countries/territories were included in the analyses, which is about 78% of all countries/territories admitted to the *ESI*. The disciplinary profiles for these 107 counties/territories were retrieved from the *ESI*, and the mean citation rates across 22 research areas were reproduced in Table 1 without any modifications. However, lowering this criterion further to 2,000 would have extended the list by 16 additional countries: Mozambique, Bolivia, Democratic Republic of Congo, Bahrein, Cambodia, Ivory Coast, Jamaica, Madagascar, Yemen, Moldova, Syria, Libya, Mongolia, Trinidad and Tobago, and Montenegro. Because the scientific strength can be measured by the number of disciplines in which a country/territory succeeded to enter *ESI*, we excluded these 16 countries as they succeeded to exceed the entrance thresholds typically only in three to four research areas and no more than in eight areas, which is <40% of the total number of research areas.

**Table 1:** The mean citation rates in 22 research fields and the average citation rate for 107 countries/territories that published 3,000 or more papers able to enter the *ESI* for the period 2009–2019.

**Country/territory**	**Agricultural sciences**	**Biology and biochemistry**	**Chemistry**	**Clinicalmedicine**	**Computer science**	**Economics and business**	**Engineering**	**Environment/ecology**	**Geosciences**	**Immunology**	**Materials science**	**Mathematics**	**Microbiology**	**Molecular biology and genetics**	**Multidisciplinary**	**Neuroscienceand behavior**	**Pharmacology and toxicology**	**Physics**	**Plant andanimal science**	**Psychiatry/psychology**	**Social sciences, general**	**Space science**	**All fields**	**Top 1%**	***INSI* (rank)**	**Dim1 (rank)**
Panama	14.7	26.0	0.0	99.2	0.0	0.0	0.0	29.6	31.3	0.0	0.0	0.0	14.3	33.5	36.5	22.9	0.0	0.0	15.2	0.0	12.9	0.0	27.3	3.7	2	68
Iceland	12.4	27.9	18.9	31.5	12.1	8.5	11.4	17.5	20.4	24.3	14.2	5.4	16.1	122.8	51.3	27.6	21.9	15.3	12.4	13.2	9.0	51.1	26.4	3.2	1	8
Switzerland	14.5	29.7	24.1	24.9	13.4	12.2	13.1	28.4	24.2	29.9	29.0	6.8	24.7	45.7	44.7	25.8	18.8	24.1	16.5	15.5	12.1	34.6	23.5	2.8	3	1
Georgia	0.0	10.7	14.8	68.3	0.0	0.0	0.0	0.0	10.1	0.0	0.0	2.2	0.0	72.9	0.0	10.5	0.0	32.0	0.0	10.1	0.0	31.1	23.1	4.2	4	98
Netherlands	17.0	25.0	23.8	25.4	10.6	14.2	12.2	24.6	24.8	26.1	25.0	5.9	26.4	44.7	30.1	26.9	18.6	23.9	18.7	18.2	12.1	32.4	22.5	2.5	5	2
Scotland	19.0	28.6	21.0	29.0	11.6	10.2	10.8	23.6	20.1	31.3	19.0	6.3	26.9	47.1	52.3	29.6	24.3	23.4	16.8	16.6	10.2	33.9	22.5	2.7	7	3
Denmark	14.2	26.3	20.5	24.5	9.5	11.6	14.7	23.2	21.5	24.3	23.0	5.7	23.4	44.1	31.4	21.8	19.3	23.2	15.8	16.1	11.5	39.4	21.5	2.5	8	10
Singapore	12.8	23.2	32.7	19.3	14.4	12.3	13.8	19.1	14.2	27.7	35.6	7.0	23.6	43.0	29.3	19.8	19.8	19.4	11.5	13.7	7.7	0.0	21.5	2.7	9	7
Wales	17.9	23.7	19.7	23.9	10.7	10.6	14.1	23.3	20.8	32.2	17.1	5.9	22.7	55.7	19.5	27.9	19.7	21.9	15.0	17.3	10.7	47.0	21.0	2.4	10	4
Estonia	10.8	25.6	15.3	41.8	4.4	5.7	7.5	23.3	12.3	19.9	12.1	4.4	20.8	64.3	20.1	18.6	20.8	24.1	17.0	14.4	6.2	25.0	20.7	2.9	6	26
Belgium	15.3	25.9	19.7	27.5	9.3	11.0	11.9	19.9	20.9	25.1	20.3	5.8	21.2	40.9	42.4	23.5	19.8	20.1	14.7	17.0	9.4	27.2	20.4	2.3	12	11
England	16.0	27.3	21.9	23.4	10.7	11.5	11.3	23.5	22.2	26.1	21.7	6.3	24.6	41.0	35.0	28.2	19.4	20.2	17.0	16.8	10.0	29.4	20.2	2.2	14	5
Ireland	18.0	24.7	21.3	21.5	9.0	9.8	12.8	19.2	18.9	33.0	28.5	5.5	25.0	46.8	40.7	29.4	19.1	19.6	13.1	14.4	8.1	40.0	19.8	2.3	16	12
Sweden	15.5	24.6	19.4	23.1	9.2	11.2	11.8	23.8	21.2	23.4	17.8	5.0	24.2	41.3	33.6	25.4	19.6	18.5	15.8	15.0	10.1	29.1	19.8	2.1	13	6
USA	13.1	26.5	24.7	19.7	11.3	14.2	11.4	19.9	20.5	26.9	28.3	6.5	24.3	37.6	32.9	25.4	18.2	19.9	13.4	16.3	9.7	26.6	19.6	1.8	21	9
N. Ireland	16.3	17.9	21.3	23.9	10.3	9.5	12.5	20.2	34.2	20.2	18.6	9.3	16.5	52.4	23.3	24.1	18.4	18.0	14.1	14.1	7.9	23.9	19.1	2.0	17	13
Austria	14.8	23.1	15.4	22.6	9.5	10.5	9.3	20.8	22.7	26.3	15.6	5.5	23.3	37.5	33.8	23.9	17.6	22.6	13.6	13.9	10.1	28.4	19.0	2.2	15	17
Zambia	0.0	0.0	0.0	36.4	0.0	12.9	0.0	16.2	0.0	19.0	0.0	0.0	17.8	0.0	32.6	0.0	0.0	0.0	8.2	9.7	8.9	0.0	18.8	2.6	40	93
Finland	15.8	23.4	16.5	23.3	10.3	11.2	10.9	20.8	20.6	22.7	15.1	6.3	20.5	46.9	21.5	25.1	18.3	20.5	14.2	14.5	8.8	29.4	18.7	1.9	23	18
Canada	14.4	23.0	19.8	23.6	11.2	12.2	11.4	19.6	17.7	23.0	18.4	5.7	21.7	33.5	25.3	22.8	17.3	19.5	13.7	15.8	10.2	34.5	18.6	1.9	25	15
Germany	12.6	23.6	19.9	19.3	9.8	9.8	9.5	20.1	19.5	25.9	19.7	5.6	21.3	34.3	30.6	22.9	17.3	18.7	15.4	13.7	8.7	29.0	18.6	1.7	27	20
Peru	9.3	13.7	9.3	36.6	0.0	5.3	7.0	21.0	20.7	20.6	9.0	3.6	17.8	39.5	21.2	14.3	10.6	23.3	9.5	13.9	7.6	13.0	18.5	2.8	11	35
Norway	14.3	20.8	14.9	24.3	10.5	10.9	11.4	21.2	20.7	22.1	13.6	6.8	17.9	42.6	35.8	24.4	16.2	19.5	14.2	13.6	10.3	38.0	18.4	2.1	18	19
France	15.1	22.6	18.2	22.0	8.8	9.0	10.2	20.7	19.5	23.9	16.9	5.8	21.1	34.5	28.4	22.3	16.9	17.0	16.3	11.7	8.4	28.8	18.1	1.7	31	22
Australia	13.7	23.6	20.7	20.4	11.4	9.1	13.2	21.3	19.5	25.5	22.1	6.2	20.8	34.6	25.2	21.5	17.5	19.2	14.7	13.9	8.7	28.0	17.7	2.0	24	16
Hong Kong	14.6	20.0	25.0	20.8	12.3	13.9	14.9	18.2	21.3	19.4	26.7	7.8	24.3	29.4	30.9	17.9	16.6	17.7	15.3	13.1	8.4	16.8	17.7	2.2	19	14
Israel	15.0	23.9	20.6	19.0	8.9	9.9	9.4	16.6	17.1	26.3	22.8	5.1	20.4	37.3	32.5	21.6	19.1	19.0	15.3	12.9	7.0	33.5	17.7	1.8	35	38
Kenya	12.4	19.5	0.0	32.3	0.0	14.4	9.9	19.5	18.2	18.2	0.0	0.0	17.3	33.3	21.4	22.3	10.4	0.0	10.3	13.8	10.8	0.0	17.7	2.2	30	21
Malawi	12.3	71.7	0.0	22.8	0.0	13.9	0.0	0.0	0.0	17.3	0.0	0.0	29.1	48.4	21.4	0.0	0.0	0.0	0.0	10.6	9.8	0.0	17.5	2.1	51	92
Luxembourg	22.8	22.9	16.9	33.6	9.1	6.6	13.7	18.3	19.3	25.1	15.4	4.2	17.2	36.4	11.0	21.7	18.9	14.7	14.3	11.7	8.8	0.0	17.4	2.3	20	23
Uganda	8.2	17.7	0.0	29.6	0.0	8.1	0.0	14.9	13.2	16.5	0.0	0.0	19.7	37.1	10.4	11.0	13.0	0.0	9.1	14.5	9.4	0.0	17.2	1.6	49	70
Italy	13.2	18.2	17.3	20.8	8.6	8.7	11.2	16.2	16.3	23.0	17.3	6.1	16.5	31.0	34.8	21.2	15.2	16.9	11.9	13.7	9.1	27.0	17.1	1.6	28	24
New Zealand	13.1	22.9	15.6	23.3	9.5	9.7	10.8	24.1	20.3	21.3	15.7	4.6	20.5	36.2	14.7	18.8	16.7	21.8	13.1	14.9	8.3	21.3	17.1	1.9	34	25
Sri Lanka	10.7	12.0	12.0	39.6	14.2	5.1	8.0	16.2	9.9	14.0	7.9	0.0	10.3	0.0	7.8	15.0	10.0	24.2	9.0	10.6	7.5	0.0	17.1	2.6	22	55
Costa Rica	11.5	11.8	0.0	32.8	0.0	8.1	0.0	21.9	19.5	22.6	0.0	0.0	11.7	82.0	21.5	21.4	14.3	0.0	9.2	13.6	10.9	0.0	16.6	1.8	45	48
Philippines	11.6	9.7	12.2	42.7	7.4	5.9	11.8	15.3	13.1	17.0	8.0	0.0	13.9	49.6	19.4	13.8	11.6	6.8	11.8	11.7	7.2	0.0	16.5	2.5	26	43
Spain	14.1	20.3	19.0	19.9	8.4	8.9	11.1	17.3	16.2	19.6	16.9	5.3	18.2	34.3	25.7	19.9	14.8	19.5	13.4	10.1	6.9	27.9	16.4	1.5	36	27
Armenia	0.0	0.0	7.4	0.0	0.0	0.0	0.0	0.0	0.0	0.0	0.0	1.4	0.0	28.8	0.0	0.0	8.7	23.1	0.0	0.0	0.0	13.2	16.3	2.7	33	28
Benin	8.8	0.0	0.0	55.5	0.0	0.0	0.0	0.0	0.0	14.8	0.0	0.0	17.5	0.0	0.0	0.0	15.9	0.0	8.5	0.0	0.0	0.0	16.3	2.2	77	100
Cyprus	12.1	12.6	17.7	23.9	10.0	8.7	12.3	20.0	21.4	0.0	14.1	5.6	0.0	20.3	0.0	14.0	9.9	30.3	10.2	10.1	6.4	18.0	16.3	2.4	29	31
Greece	13.2	18.7	17.4	19.7	9.3	8.2	12.3	14.3	15.5	25.8	14.5	5.6	17.0	37.4	28.4	16.6	15.1	20.5	11.0	11.6	10.3	21.8	16.3	1.7	54	105
Tanzania	9.6	18.2	0.0	25.1	0.0	8.4	0.0	13.8	13.0	19.1	0.0	0.0	20.9	32.9	19.9	13.0	11.8	0.0	8.5	9.4	10.4	0.0	16.0	1.6	52	67
Portugal	14.2	18.4	16.6	16.4	7.5	8.7	12.1	16.4	16.2	22.7	16.5	4.8	17.3	28.5	20.8	22.5	16.6	21.0	12.1	9.2	7.0	30.8	15.4	1.5	39	29
Hungary	8.0	16.1	12.3	20.5	6.3	6.0	6.8	14.3	11.2	22.4	10.2	3.4	13.4	32.1	21.9	17.5	13.3	22.4	10.1	14.8	7.1	25.4	14.8	1.7	50	41
Uruguay	12.3	17.2	13.8	36.9	4.5	3.6	9.3	18.4	13.5	14.9	0.0	3.2	12.1	17.0	80.1	17.4	11.8	9.4	9.0	20.5	4.5	0.0	14.8	1.3	38	33
Botswana	0.0	0.0	0.0	53.3	0.0	0.0	0.0	10.3	8.6	15.6	0.0	0.0	0.0	0.0	65.2	0.0	0.0	0.0	0.0	0.0	5.5	0.0	14.3	1.4	99	106
Qatar	0.0	11.8	14.5	19.7	11.4	4.9	10.6	9.4	0.0	17.4	13.9	5.0	0.0	30.4	41.9	11.8	7.6	17.4	0.0	5.6	4.8	15.5	14.0	2.3	37	63
Zimbabwe	15.1	0.0	0.0	32.5	0.0	0.0	0.0	12.0	9.3	16.3	0.0	0.0	0.0	0.0	0.0	0.0	0.0	0.0	8.7	7.9	8.1	0.0	13.8	1.8	91	94
Japan	7.8	15.1	15.8	12.9	5.8	5.3	7.3	13.3	15.8	21.8	15.5	3.8	14.3	28.6	22.2	15.7	10.9	12.9	10.6	9.6	7.8	22.6	13.6	0.9	102	97
Sudan	6.9	0.0	7.6	33.2	0.0	0.0	11.8	0.0	17.5	15.4	0.0	0.0	9.8	0.0	0.0	0.0	8.3	0.0	6.2	0.0	0.0	0.0	13.6	1.0	62	37
Ghana	7.0	10.0	8.6	29.9	0.0	5.1	7.3	12.4	10.3	13.8	0.0	0.0	15.5	28.1	8.6	11.1	10.2	0.0	5.9	7.1	7.6	0.0	13.5	1.6	61	83
Slovenia	11.4	21.8	12.2	14.0	7.7	6.2	7.8	15.2	11.1	21.7	11.0	4.6	19.3	32.5	21.5	19.6	13.1	25.5	10.1	12.2	4.1	53.6	13.5	1.4	44	30
Saudi Arabia	9.6	14.1	17.7	12.5	9.4	5.5	10.6	14.9	11.2	16.3	18.3	6.8	10.9	34.2	15.4	12.2	8.4	13.2	11.3	10.6	6.7	15.1	13.4	2.3	32	34
South Africa	8.4	16.2	12.0	23.0	8.5	4.4	9.3	15.1	13.7	19.3	10.6	4.4	16.6	25.7	13.8	17.2	11.2	16.4	10.1	9.1	6.7	31.1	13.4	1.6	42	36
Lebanon	11.3	10.8	10.0	19.8	5.8	10.3	8.8	10.1	7.4	16.3	11.3	2.5	10.7	20.3	0.0	11.9	12.3	8.8	8.0	23.8	9.2	45.1	13.3	1.8	41	49
Czech Republic	8.4	13.5	13.2	22.7	5.4	3.8	6.7	16.3	10.9	17.0	10.0	4.2	13.3	25.8	14.9	18.0	13.5	16.3	10.5	8.5	4.6	17.0	13.2	1.3	83	85
Nepal	9.5	15.4	0.0	16.4	0.0	8.1	10.1	12.6	20.0	14.5	19.2	0.0	0.0	0.0	0.0	0.0	11.7	0.0	6.6	10.7	7.8	0.0	13.2	1.6	48	44
Chile	9.2	11.6	9.5	16.5	7.4	4.9	9.9	12.9	15.2	11.2	8.8	5.0	11.9	27.3	16.1	17.4	12.2	16.7	8.5	7.0	4.5	27.6	13.1	1.4	47	45
Malta	0.0	23.0	0.0	0.0	0.0	0.0	0.0	0.0	0.0	0.0	0.0	0.0	0.0	62.1	0.0	16.6	12.5	0.0	0.0	6.1	4.9	24.3	13.0	1.8	94	102
Argentina	10.6	12.6	10.9	22.6	5.7	5.8	8.8	14.7	10.8	18.0	10.7	4.0	10.6	19.6	18.0	18.9	10.9	17.0	8.9	9.4	4.9	15.1	12.8	1.1	56	46
Burkina Faso	7.9	0.0	0.0	0.0	0.0	0.0	17.1	0.0	0.0	17.7	0.0	0.0	15.8	27.4	21.9	0.0	0.0	0.0	8.7	0.0	8.9	0.0	12.5	1.0	104	96
Latvia	10.0	13.2	9.0	28.7	0.0	7.4	4.4	16.0	0.0	19.7	8.8	0.0	0.0	74.4	0.0	0.0	9.7	8.3	8.0	0.0	6.8	0.0	12.5	1.9	72	90
Bulgaria	6.0	6.6	9.3	20.0	4.9	6.8	7.6	12.4	11.6	16.0	8.6	3.7	12.1	37.7	1.4	14.7	8.7	23.0	7.3	25.9	6.9	14.0	12.4	1.5	73	32
Taiwan	13.1	14.5	15.2	13.2	8.1	7.1	9.0	11.2	14.2	13.6	15.7	4.8	12.7	18.6	14.4	13.8	13.5	14.0	10.9	9.6	7.5	28.1	12.4	0.8	46	54
Colombia	7.9	10.9	10.2	20.3	6.8	5.8	6.1	16.4	13.4	16.3	8.9	2.8	12.5	27.4	12.9	14.9	8.4	21.2	7.0	11.1	5.5	14.9	12.3	1.7	43	52
South Korea	8.3	14.7	16.7	11.6	6.1	6.6	7.6	10.5	13.7	15.1	16.7	4.5	10.8	19.1	17.6	13.9	13.0	12.6	9.9	9.4	6.9	18.4	12.2	0.9	71	40
Belarus	0.0	10.0	5.5	0.0	0.0	0.0	3.7	0.0	0.0	0.0	5.3	2.4	0.0	24.9	0.0	0.0	11.0	20.7	0.0	0.0	0.0	0.0	12.0	2.0	92	99
Cameroon	7.3	10.2	7.9	23.8	0.0	4.1	7.7	17.1	7.3	12.7	9.6	3.4	14.7	32.7	15.0	13.6	10.0	5.8	7.6	0.0	7.1	0.0	11.9	1.3	70	77
Oman	9.4	11.7	12.0	23.2	5.1	4.3	9.7	10.3	9.4	0.0	9.1	3.3	15.3	21.7	0.0	12.2	10.3	10.1	11.6	8.9	0.0	0.0	11.9	1.5	74	76
Bangladesh	8.2	9.9	9.2	28.8	5.6	5.1	9.1	13.2	9.9	18.1	7.1	0.0	15.5	22.5	8.8	13.0	10.4	7.2	6.1	8.2	8.9	0.0	11.8	1.4	67	47
Croatia	10.1	12.5	11.0	13.5	5.2	3.1	5.6	11.0	10.1	18.2	7.3	3.7	14.4	59.1	42.3	16.2	11.1	23.4	6.0	8.4	3.6	23.6	11.8	1.3	60	69
Thailand	9.6	12.4	11.6	15.3	6.3	6.4	10.3	10.9	12.0	17.8	10.8	3.9	13.4	19.9	20.2	14.6	11.9	13.8	8.7	9.5	7.0	13.3	11.8	1.0	55	51
China	10.2	12.1	15.5	9.9	8.0	7.3	8.9	11.2	12.0	12.8	15.7	5.0	10.3	14.2	17.1	12.3	10.3	10.2	10.0	8.4	8.3	14.0	11.7	1.2	98	91
Indonesia	7.7	13.2	10.0	27.0	4.6	5.1	9.6	17.0	12.1	15.0	7.2	3.1	15.5	14.5	14.9	12.8	8.1	5.2	8.1	7.2	7.7	0.0	11.7	1.2	66	62
Senegal	9.1	0.0	0.0	0.0	0.0	6.2	0.0	12.0	13.8	18.2	0.0	3.0	14.2	26.7	34.4	0.0	0.0	0.0	10.5	0.0	6.8	0.0	11.7	1.2	59	42
Venezuela	5.5	17.8	13.1	15.2	9.3	1.9	6.5	15.1	16.2	19.3	8.3	3.3	14.8	40.0	44.5	13.8	9.9	8.9	7.1	15.8	6.5	20.3	11.6	1.1	65	39
UAE	8.4	11.4	15.2	18.6	6.5	5.9	9.4	14.7	9.7	19.4	10.5	4.5	13.9	22.8	20.7	16.5	9.8	7.6	8.2	6.1	5.7	13.0	11.3	1.4	53	50
Ecuador	7.0	11.6	6.3	21.6	5.2	4.0	5.7	16.0	13.4	25.4	10.8	0.0	11.0	16.9	0.0	12.4	9.3	13.9	7.3	0.0	4.4	0.0	11.2	1.8	57	53
Malaysia	10.0	13.1	12.5	12.7	8.2	5.4	13.3	12.5	11.0	14.6	10.7	4.7	10.7	14.3	4.6	10.3	10.1	11.5	7.6	8.4	6.0	13.3	11.2	1.3	63	80
Slovakia	7.6	10.2	10.0	19.4	5.3	2.5	5.6	12.2	8.5	17.4	6.4	4.6	10.7	26.1	15.1	16.2	14.1	16.4	8.8	5.0	6.9	10.4	11.1	1.1	69	58
Azerbaijan	0.0	0.0	3.6	0.0	8.2	0.0	5.3	0.0	0.0	0.0	3.5	2.4	0.0	0.0	0.0	0.0	0.0	20.8	0.0	0.0	0.0	0.0	11.0	2.0	100	103
Ethiopia	7.2	7.4	6.7	19.9	0.0	10.6	7.3	8.9	13.0	11.1	0.0	5.3	9.4	23.0	5.8	12.2	10.3	0.0	7.0	9.7	8.3	0.0	11.0	1.3	64	88
Macau	0.0	19.8	11.9	0.0	11.3	7.4	9.4	0.0	0.0	0.0	17.0	3.3	0.0	45.1	22.7	7.0	10.6	0.0	0.0	6.2	4.9	7.2	11.0	2.3	80	79
Cuba	7.1	12.2	8.5	0.0	7.5	0.0	7.0	10.4	12.1	16.3	8.5	0.0	17.3	0.0	0.0	16.4	8.8	16.8	6.7	8.4	7.0	0.0	10.6	0.8	97	82
Mexico	8.7	12.1	9.6	18.4	6.2	4.8	7.0	10.7	10.5	14.9	8.0	3.6	11.2	21.4	21.7	11.9	10.1	13.2	7.4	9.7	5.7	17.7	10.6	1.0	76	57
Lithuania	6.2	12.9	10.4	13.9	6.1	6.5	4.9	10.4	10.4	25.6	6.5	2.9	14.4	45.1	0.0	11.5	10.6	17.0	6.1	11.2	5.5	18.5	10.4	1.3	68	56
India	6.1	11.7	12.3	11.4	5.8	6.4	8.6	11.3	9.5	12.4	11.0	4.0	10.2	13.7	6.8	11.0	11.2	10.1	7.7	9.1	6.3	15.4	10.3	0.7	79	65
Morocco	9.4	9.9	9.9	12.3	4.3	5.1	6.6	9.1	11.8	21.0	9.5	3.3	10.2	15.4	0.0	7.3	11.2	16.5	7.7	11.7	8.9	21.4	10.3	1.1	78	60
Poland	8.3	11.3	9.8	15.1	6.4	3.5	6.0	8.2	8.3	17.1	8.0	3.8	9.1	19.0	11.2	13.6	10.6	12.8	6.7	8.6	5.7	26.1	10.3	0.9	86	64
Vietnam	8.1	10.4	8.5	31.6	7.4	4.2	7.3	9.8	11.4	19.3	8.1	3.4	18.1	22.2	5.9	13.6	7.7	9.4	7.1	6.3	8.4	16.0	10.0	1.5	58	61
Jordan	7.4	9.6	8.8	18.2	5.6	6.4	8.0	7.2	9.8	14.9	8.4	7.0	0.0	24.6	0.0	12.8	7.0	6.8	6.3	7.6	5.1	0.0	9.9	1.2	85	81
Kuwait	10.4	11.8	11.3	11.5	9.9	4.3	8.9	9.8	8.1	21.2	9.5	3.3	10.0	24.1	0.0	9.7	10.3	0.0	0.0	6.8	5.4	0.0	9.9	1.4	82	78
Brazil	6.3	11.8	10.9	11.8	6.2	4.3	7.8	11.7	11.4	14.6	9.2	4.2	11.1	14.7	10.1	14.2	10.1	13.3	6.3	10.9	5.4	20.5	9.8	0.7	87	59
Serbia	8.1	6.6	8.6	10.8	5.3	2.8	5.9	8.2	9.9	15.8	8.4	6.8	10.2	28.4	0.0	13.7	9.4	21.0	5.7	9.2	3.7	9.6	9.6	1.1	81	73
Egypt	7.3	8.3	10.6	10.5	5.4	6.6	8.4	9.4	6.7	12.7	10.8	5.7	8.7	13.4	33.6	11.3	9.3	12.0	7.1	8.4	6.3	5.7	9.5	0.8	88	66
Iran	8.3	8.8	11.7	8.5	7.5	6.6	9.3	8.7	8.4	8.7	11.7	4.2	6.0	10.0	4.6	9.8	9.2	9.3	5.5	6.9	5.3	12.1	9.2	0.8	89	75
Pakistan	7.5	10.0	8.9	12.1	6.0	4.6	8.6	10.5	8.3	16.7	8.4	5.1	11.1	23.7	4.9	12.6	5.9	12.5	5.4	7.4	6.5	10.2	9.0	1.2	75	71
Nigeria	6.1	7.9	9.3	14.3	4.1	4.7	7.7	6.9	5.1	11.4	8.1	3.0	9.4	33.7	8.4	11.0	6.5	6.5	5.4	13.6	4.6	6.0	8.8	1.0	84	84
Ukraine	9.5	11.5	7.4	46.6	4.2	0.7	4.1	10.6	8.0	13.0	4.7	2.8	7.3	12.3	12.6	6.3	10.9	9.9	8.6	13.0	6.6	11.2	8.6	0.9	90	86
Tunisia	11.2	10.0	8.3	11.6	4.2	7.0	7.0	10.7	7.8	13.3	8.5	3.5	9.2	12.3	13.1	8.5	10.8	6.9	8.5	8.2	7.6	0.0	8.5	0.5	96	74
Turkey	8.3	8.8	11.0	6.8	8.8	6.7	10.0	6.8	10.6	15.7	9.4	4.6	6.8	19.6	4.9	8.3	8.6	13.0	5.1	5.7	4.0	15.0	8.3	0.6	93	72
Algeria	8.1	8.9	7.5	28.4	5.0	0.0	8.5	6.3	7.5	12.3	8.7	3.8	10.2	0.0	0.0	15.8	6.9	6.1	7.5	0.0	0.0	32.6	8.1	0.8	101	89
Iraq	5.7	6.8	8.0	22.6	4.1	0.0	7.0	0.0	4.8	0.0	6.5	3.8	0.0	0.0	0.0	8.4	5.8	4.7	0.0	28.0	0.0	0.0	7.9	1.1	103	95
Bosnia and Herzeg	0.0	0.0	0.0	0.0	6.2	0.0	4.2	0.0	0.0	0.0	0.0	2.6	0.0	0.0	0.0	0.0	0.0	16.7	0.0	5.7	0.0	0.0	7.6	1.2	106	104
Russia	6.7	9.7	6.2	16.0	2.9	4.5	4.0	7.9	6.8	16.1	6.0	2.8	10.1	13.9	14.4	7.3	7.8	8.6	6.5	4.4	3.5	13.6	7.6	0.6	95	87
Uzbekistan	0.0	0.0	3.6	0.0	0.0	0.0	0.0	0.0	0.0	0.0	7.3	3.1	0.0	0.0	0.0	0.0	0.0	6.3	0.0	0.0	0.0	18.0	6.7	0.5	107	107
Kazakhstan	0.0	7.7	4.4	0.0	5.3	0.0	3.3	0.0	7.0	0.0	4.9	2.4	0.0	0.0	33.9	0.0	0.0	8.1	0.0	0.0	0.0	14.5	6.5	0.7	105	101

**Figure 1:**
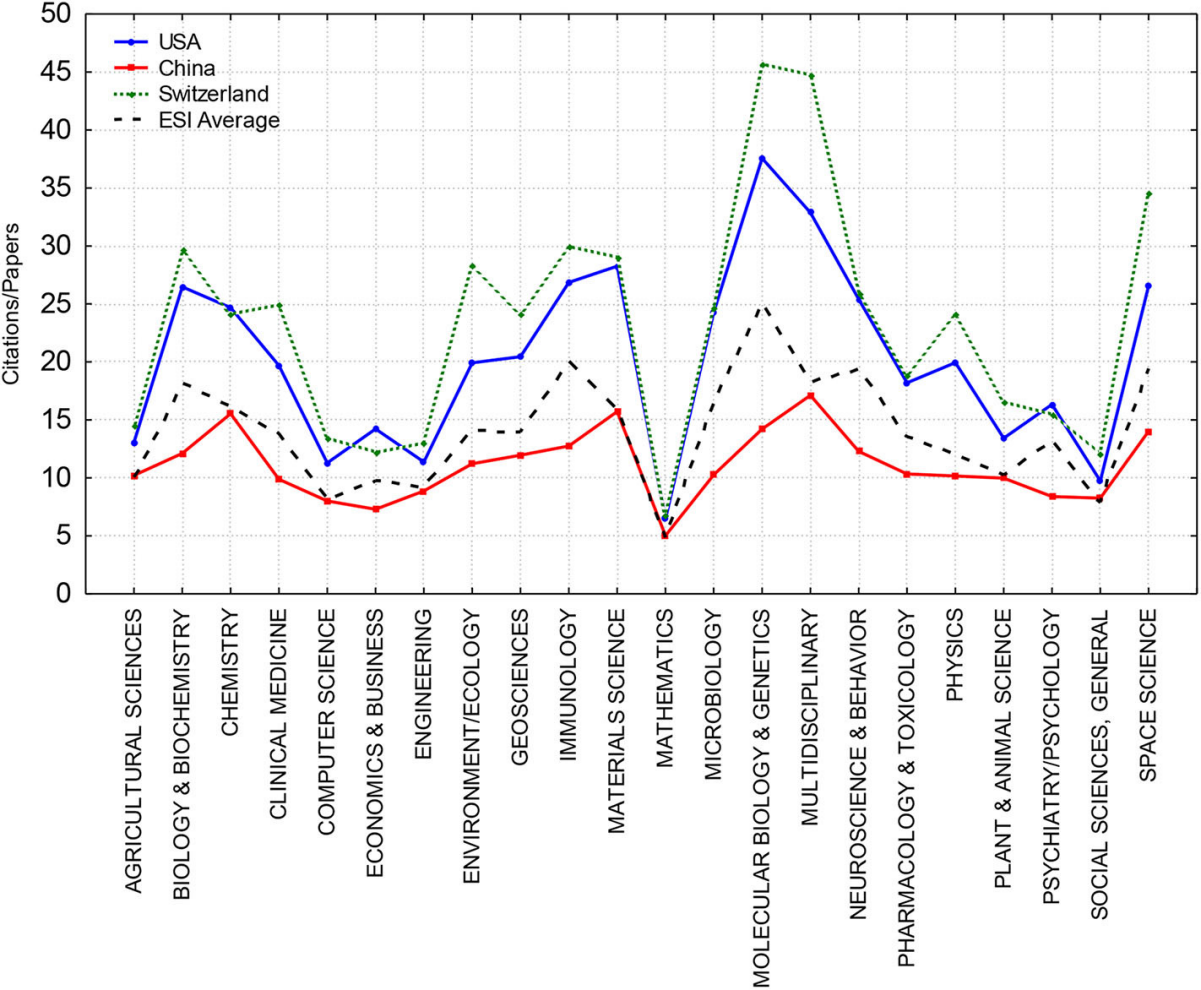
The mean citation rates across 22 research fields for three countries, USA (blue), China (red), and Switzerland (green), compared with the *ESI* average (black broken line).

*Zeros indicate research fields in which a country/territory did not exceed required number of citations needed to enter the ESI. The penultimate column shows the Indicator of a Nation's Scientific Impact (INSI) ranking, which, in equal parts, takes the mean citation rate, percentage of papers reaching top 1% citation rate, and the number of research fields that have passed the entrance threshold. The last column shows ranking on the x-axis (Dim1) of Figure 1. Countries are ranked according to the mean citation rate (the 4th column from the right “All fields”)*.

### Indicator of a Nation's Scientific Impact (*INSI*)

The penultimate column [“*INSI* (rank)”] in Table 1 presents the country/territory *INSI* score (ranking), which is an average of three components (Allik et al., 2020b). The first component is the country/territory mean citation rate—the number of citations divided by the number of papers (the 4th column from the right “All fields”). The second component is the percentage of papers that had reached the top 1% citation rate in the respective research area and age cohort (the 3rd column from the right “Top 1%”). Finally, the third component is a number of research areas or disciplines in which each country/territory had reached the *ESI* (the number of nonzeros in the first 22 columns). For example, large countries such as USA, Germany, China, and Russia have surpassed the *ESI* entrance thresholds in all 22 research fields. However, 49 (46%) out of 107 countries/territories failed to reach the *ESI* in one research area at least. Before computing the average score, three *INSI* components were normalized so that their mean values were equal to zero with the standard deviation equal to one. Thus, the *INSI* scores in the last column are in the units of the standard deviation showing how much below or above the average score of all 107 countries/territories each participant was scoring.

Table 1 also reproduces the mean citation rates of each country/territory in 22 different research fields. Zeros represent research fields in which country/territory failed to enter the *ESI*. For example, Benin, Bosnia, and Herzegovina, and Uzbekistan had 16 zeros in their disciplinary profiles. Because no entry means no citations, we treated those research areas as if they had zero citation rates.

For the analysis of 107 disciplinary profiles of each country/territory across 22 different research fields, we used the MDS technique, which attempts to transform “distances” or “proximities” among a set of *N* objects into a configuration of *N* points mapped into a geometric space with the smallest possible number of dimensions. A non-metric version of MDS assumes that only the ranks of the distances are known or relevant for producing a map, which reproduces these ranks in the best possible way. We applied the non-metric Guttman-Lingoes MDS algorithm (Borg and Groenen, 2005) as it is implemented in the *Statistica* (*Dell* Inc.) software package. Before applying a MDS algorithm, a matrix of pairwise (dis)similarities between disciplinary profiles of any two countries/territories was computed. The absolute pairwise differences across 22 disciplines were summed together, being used as a measure of (dis)similarity between any pairs of countries/territories. As a result, we created a symmetric matrix with 11,449 elements, each of which showing City Block or Manhattan distance between all possible pairs of countries/territories including the main diagonal of zeros representing distance from oneself. In order to compress the large range of differences in these (dis)similarities, we normalized (the mean value became zero with the standard deviation one) sums of absolute differences across 107 countries/territories.

## Results

Table 1 presents the mean citation scores across 22 research fields for each country/territory. Entries in the table are ranked according to the mean citation rate (the 4th column from the right “All fields”). Panama (27.3), Iceland (26.4), and Switzerland (23.5) had the highest mean citation rate (note that fields with zeros were not used for the calculation of the mean citation rate). Among 107 countries/territories, publications authored by Russian (7.6), Uzbekistan (6.7), and Kazakhstan (6.5) scientists had the smallest impact on the world science in terms of cited work.

To obtain a better impression about the disciplinary profiles, Figure 1 displays the mean citation rates across 22 research areas for Switzerland, USA, and China. These three nations were chosen for illustrative purposes only: China and USA were two the most prolific sciences in the world publishing over 2 million and 4 million papers, respectively, during the observed 11-year period; Switzerland was one of the most efficient sciences by the mean citation rate. Please notice that these three nations entered the *ESI* in all 22 disciplines. The *ESI* average citation rate in each research area is also shown as a black broken line providing a baseline with which each nation can be compared. Switzerland, as a long-time efficiency front-runner, has a higher citation rate than USA in almost all research areas. Although the impact of Chinese science is growing (Leydesdorff and Wagner, 2009; Leydesdorff et al., 2014), its mean citation rate is still below the *ESI* average in almost every 22 research fields.

The penultimate column in Table 1 presents the *INSI* ranking for each country/territory. According to this ranking, the highest-quality science is produced in Iceland, Panama, Switzerland, Republic of Georgia, Scotland, Estonia, Netherlands, Singapore, Denmark, and Wales. As these are all relatively small countries, this confirms previous findings that small countries seem to have an advantage in publishing high-impact scientific papers (Allik et al., 2020a,b). According to the *INSI*, the smallest impact among these 107 countries/territories had publications authored by researchers from Iraq, Burkina Faso, Kazakhstan, Bosnia and Herzegovina, and Uzbekistan. It is also interesting to notice that China, in spite of the increasing research volume, occupied a position in the middle of the *INSI* ranking (the 59th position) and Russia was very close to the bottom (the 95th position).

Next, we were interested in how the disciplinary profiles of the mean citation rates were related to the overall scientific impact of countries/territories. In a previous study (Bongioanni et al., 2014), a complex index, borrowed from the physics of magnetism, was proposed to estimate overlaps between disciplinary profiles of countries. In this study, we preferred a simpler approach computing the sum of absolute differences across all 22 fields between two disciplinary profiles (see *Methods* section). The findings showed that a two-dimensional solution was optimal (stress function = 0.098), showing that all differences between countries/territories can be placed on a plane with a sufficient accuracy (cf. Mair et al., 2016).

**Figure 2:**
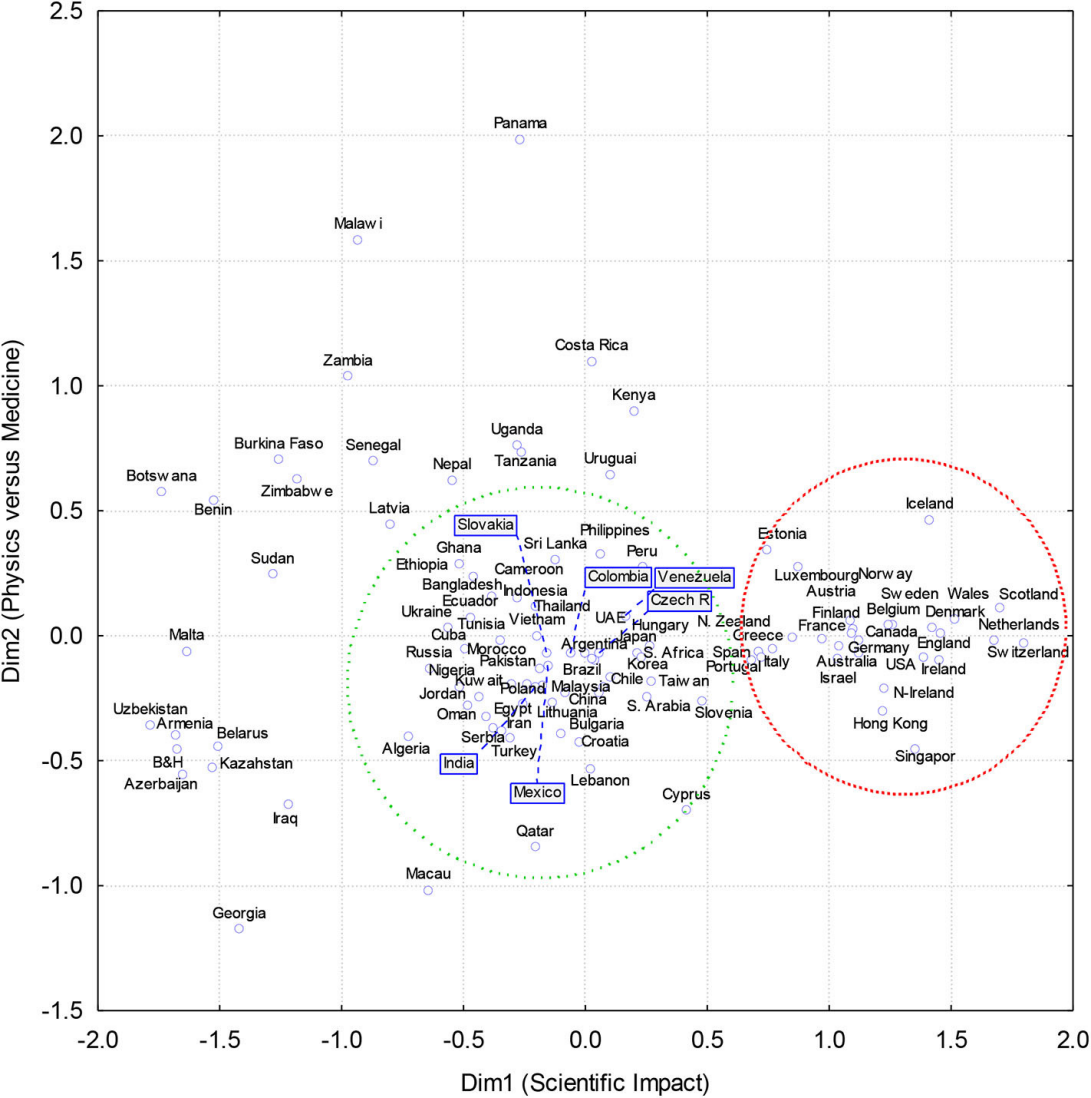
Two-dimensional plot of a non-metric Guttman-Lingoes multidimensional scaling analysis of country's citation profile similarities (Manhattan or City Block metrics of the normalized citation rates).

Figure 2 shows a two-dimensional plot derived from the MDS of similarities–differences between the disciplinary profiles of the mean citation rates. The first dimension Dim1 can be identified as the country/territory's overall scientific impact. Rankings of countries/territories on this dimension Dim1 is presented in the last column in Table 1 [“Dim1 (rank)”]. The correlation between Dim1 and the *INSI* was *r* = 0.81 (*N* = 107, *p* < 0.0001), which is higher than correlations between Dim1 and any of the *INSI* three components: the mean citation rate (*r* = 0.64, *p* < 0.00001), the percentage of the top-cited papers (*r* = 0.35, *p* < 0.00001), and the number of areas represented in the *ESI* (*r* = 0.77, *p* < 0.00001). After excluding two largest outliers—Panama and Georgia—the correlation increases to *r* = 0.88. Thus, this indicated that the scientific impact of nations could be measured using the Dim1 scores with approximately the same accuracy as with the *INSI*. It is important to emphasize that this ranking was obtained by ignoring the absolute mean citation rates, which is the foundation of the *INSI*. When a transformation for the pairwise differences AB, AC, and BC between any triples of the disciplinary profiles A, B, and C were searched to satisfy an approximate equality AB + BC ≈ AC, information about the absolute elevation of profiles was lost. Because the triangulation rule was sustained with a reasonable accuracy, it indicated that all differences between profiles can be arranged on a linear scale.

To illustrate how this derived ranking of the scientific impact [Table 1, the last column “Dim1 (rank)”] has certain advantages before the previous ones, we need to observe changes in the ranking positions of countries, whose high positions may not be entirely justified. According to the mean citations rate, by which countries/territories were listed in Table 1, Panama (27.3) was number one in the world, Georgia (23.1) was on the 4th position and Peru (18.5) occupied the 22nd position. Because the *INSI* penalizes for failures to reach the *ESI* in any of the research areas, countries/territories not being successful in all 22 research areas were expecting to lose positions in the ranking. Because both Georgia and Panama did not enter the *ESI* in 11 research areas, they were shifted down in the *INSI* ranking to the 2nd and the 5th positions, respectively. At the same time, Peru did not reach the *ESI* only in one field (computer sciences); the position in the *INSI* ranking was elevated up to the 12th position.

Compared with these relatively small changes in the ranking of countries that was based on either the mean citation rate or the *INSI*, the differences in the countries' ranking positions on Dim1 derived from the MDS analysis were more substantial. According to their positions on Dim1 (the last column in Table 1), Georgia, Panama, and Peru occupied the 98th, 68th, and 35th positions, respectively. Thus, in comparison with the mean citation rate, Georgia, Panama, and Peru dropped 94, 67, and 13 positions. Their disciplinary profiles were more similar to the disciplinary profiles of nations in vicinity to these positions. Two countries with profiles the most similar to Panama in this new ranking were Tanzania and Bangladesh. Georgia was squeezed between Sudan and Belarus, not Belgium and Ireland as previously in the *INSI* ranking. These changes in the ranking positions explained, as was already mentioned, a relatively modest correlation between Dim1 and the mean citation rate (*r* = 0.64).

The second dimension Dim2 was more difficult to interpret because a clear pattern did not emerge. Because it has the largest positive correlations with the mean citation rate in Clinical Medicine (*r* = 0.49, *p* < 0.00001) and Social Sciences (*r* = 0.50, *p* < 0.00001) to the contrast negative correlations with the mean citation rates in Physics (*r* = −0.49, *p* < 0.00001) and Mathematics (*r* = −0.49, *p* < 0.00001), it would be fair to say that this dimension represents human-centered opposite to the physics–math-centered sciences.

Two distinct clusters can be identified on the plot. These two clusters, we need to warn, were identified based on an impression with a heuristic purpose only, not in the result of any rigorous procedure. The first cluster (surrounded by the red circle) represents the cream of the crop in the world of science. This cluster of 29 countries includes mainly European countries such as the Netherlands, Scotland, and Switzerland but also other countries such as USA, Singapore, Hong Kong, Canada, Australia, Israel, and New Zealand. Although it was noticed that the scientific wealth of Hong Kong and Singapore is declining (Horta, 2018), they firmly belong to this group of leaders in the world science. A common feature of these 29 countries/territories is that they all succeeded to pass the *ESI* entrance thresholds in all 22 research areas.

Another group (green circle) unites not only many of the world's largest countries—China, Russia, Brazil, and India—but also smaller countries like Slovenia, Ecuador, and Hungary. If large countries in this cluster were successful in all 22 disciplinary areas, then smaller countries may have difficulties to collect enough citations to exceed the *ESI* entrance thresholds in some research areas. Outside of these two groups (or circles) are mainly African countries (upper part) or post-communist countries (lower part), which scatter along Dim2.

A similarity between these two clusters and two clusters that were identified previously (Bongioanni et al., 2015, Figure 2) can be noticed. Bongioanni et al. (2015) identified a cluster that included countries with a prominent biomedical disciplinary profile such as the US and the Netherlands (Bongioanni et al., 2015). Another cluster embraced a group of countries with a conspicuous physical-sciences profile, like China and Russia. In addition, many Central, Southern, and Eastern European countries belonged to this second group, as well as India, Indonesia, and Mexico. However, there are notable differences between the findings of Bongioanni et al.'s (2015) and this study. According to Bongioanni et al. (2015), Turkey is in the same group with the UK and the Netherlands; in the current study, Turkey's nearest neighbors are Serbia and Iran in the second group. In addition, Estonia and Portugal were differently classified. According to Bongioanni and colleagues (2005), these two countries are in the less scientifically advanced group of nations, while in our classification, they more likely belong to the leading group science nations (Bongioanni et al., 2015). These discrepancies are probably produced by different measures of (dis)similarity between disciplinary profiles.

## Discussion

It has been suggested by experts that new impact indicators should not be introduced unless they have a clear added value relative to the existing indicators (Waltman, 2016). Indeed, the average citation rate or the percentage of papers reaching the top of the citation distributions have proved to be trusted and reliable indicators of the scientific wealth of nations (May, 1997; Rousseau and Rousseau, 1998; van Leeuwen et al., 2003; King, 2004; Halffman and Leydesdorff, 2010; Prathap, 2017). Very serious arguments are needed to introduce yet another indicator. Although warnings are still released not to take citations as the only constituents of the concept of scientific quality (MacRoberts and MacRoberts, 2018; Aksnes et al., 2019), citation indicators have become the most convenient measures of the scientific strength of nations (May, 1997; Rousseau and Rousseau, 1998; King, 2004; Harzing and Giroud, 2014; Prathap, 2017). Nevertheless, some of the country rankings based on the citation statistics did not look credible (Allik, 2013; Allik et al., 2020a,b). One of the possible causes of these counterintuitive rankings, as was mentioned above, appears to be the selectivity of databases, which is the main tool for extracting what is believed to be essential in science (Allik et al., 2020a,b). Although it appears to be true that the top of the citation distribution is a more informative characteristic of the scientific impact than indicators based on average values (van Leeuwen et al., 2003), the selectivity of databases unwillingly eliminates “losers” whose counting would have decreased the mean citation rate. Thus, the scientific impact of nations can be increased not only by the number of highly cited papers but also by neglecting those papers that could jeopardize the mean citation rate (Allik et al., 2020a). To improve citation indicators, a new measure—*INSI*—was proposed, which, in addition to the citation statistics, takes also into account the number of research areas in which a country/territory was successful to enter the *ESI*. This amendment improved rankings in the right direction, unfortunately not radically enough (Allik et al., 2020b). As we said above, the disciplinary profiles appeared to be different for scientifically developed and non-leading countries (Kozlowski et al., 1999; Almeida et al., 2009; Yang et al., 2012; Bongioanni et al., 2014, 2015; Harzing and Giroud, 2014; Carley et al., 2017; Li, 2017; Daraio et al., 2018; Shashnov and Kotsemir, 2018). For example, it was noticed that one of the reasons why post-communist countries are still lagging behind Western counterparts is their archaic disciplinary structure reflecting, among other things, the demands of the former totalitarian regimes (Kozlowski et al., 1999; Markusova et al., 2009; Jurajda et al., 2017). Openness of national science systems was observed to be correlated with the scientific impact—the more internationally engaged a nation is, in terms of coauthorships and researcher mobility, the higher the impact on their scientific work (Wagner et al., 2018). It was noticed that geographical proximity, which is one of the strongest incentives for cooperation, may be a principal factor of the similarity between disciplinary profiles (Almeida et al., 2009). Although such pairs as Finland–Norway, England–Scotland, Netherlands–Belgium, and Denmark–Sweden (Almeida et al., 2009, Figure 4) support this idea, there is an equally large number of neighboring countries (e.g., Panama–Colombia, Peru–Ecuador, Georgia–Armenia, Estonia–Latvia, etc.) that have a distinctly different level of scientific impact. It was also observed that BRICS countries differ from the scientifically leading countries typically belonging to G7 not only by the overall scientific impact but also by differences in the disciplinary structure of their sciences (Bornmann et al., 2015; Li, 2017; Shashnov and Kotsemir, 2018; Yue et al., 2018). For example, it was observed that a competitive advantage of a group of nations including the Netherlands, USA, UK, Canada, and Israel is an emphasis on social and biomedical research (Harzing and Giroud, 2014). The disciplinary citation profiles of G7 and BRICS countries are noticeably different. For instance, most G7 countries performed well in Space Science, which was not the strength of BRICS countries (Shashnov and Kotsemir, 2018; Yue et al., 2018). In spite of these differences, there seems to be a common evolutionary pattern of convergence in the national disciplinary profiles (Bongioanni et al., 2014; Bornmann et al., 2015; Li, 2017).

Typically, the disciplinary profiles were analyzed to discover different clusters into which nations belong. Another approach, adopted in this study, was to see if there is a small number dimensions that can summarize (dis)similarities between the disciplinary profiles (cf. Borg and Groenen, 2005). It is not likely that the similarities and dissimilarities between disciplinary profiles have a distinct pattern, which could be described by a low-dimensional space. Like any other human enterprises, science is a complex institution, which may have differences in prioritizing various research fields. For example, Panama in collaboration with the Smithsonian Institution—one of the world's largest museum, education, and research complexes—invested into the study of the tropical ecosystems by creating a branch of the Smithsonian in Panama, which attracted the best researchers around the world in this area (cf. Rubinoff and Leigh, 1990). Another already mentioned example is Georgia allocating considerable assets into physics in order to develop partnerships with the large international collaborative networks. As a result, Georgia achieved the highest mean citation rate (on average 32 cites per paper) in physics (see Table 1, column “Physics”). Inspecting Table 1, one can also notice, with a surprise, that Kenya had the highest impact among 107 nations in economics and business: every paper that was published by Kenyan's economists collected 14.4 citations on average (column “Economics and business”). Kenya benefited from the research unit of the United Nations Environment Programme in Nairobi, which is devoted to the study of the economics of ecosystems management and provided services (cf. Ivanova, 2007). Knowing the accomplishments that the deCODE and Kári Stefánsson with his colleagues (Hakonarson et al., 2003) have achieved, it is not surprising that Iceland seized the first position in the impact ranking in the molecular biology and genetics (column “Molecular biology and genetics”). These examples seemed to suggest that nations might have different keys for their success in producing high-quality science.

Nevertheless, all (dis)similarities between disciplinary profiles can be arranged on a single dimension ranking, which corresponded to the scientific impact that was measured by conventional indicators such as the *INSI*. This demonstrated that in spite of differences in the nations' competitive advantages, all that mattered was overall impact across many disciplines as possible, not how this impact was allocated among various research areas. To attain success, it was essential to have an evenly high level of citations relative to the *ESI* average across as many disciplines as possible because low impact or not even exceeding the entrance thresholds in one or several research areas is a key factor that diminishes scientific impact. This may also demonstrate that attempts of the agencies that fund scientific research in prioritizing their disciplinary budgets are not as effective as usually claimed. Results of this study appeared to suggest that the only thing that was really worth prioritizing is the scientific excellence irrespective of which particular discipline it was demonstrated. To our satisfaction, the impact ranking derived from the (dis)similarities between disciplinary profiles was free from anomalies that traditional citation indicators typically possess. These results support an idea about a common route toward scientific excellence in which disciplinary peculiarities are supporting a general advancement (Bongioanni et al., 2014; Li, 2017; Thelwall and Levitt, 2018).

In conclusion, previous attempts to construct indicators of the scientific impact of nations were based on the average or the top-citation statistics. However, the country rankings based on these indicators often look problematic and counterintuitive. Most of these anomalies were produced by failures to exceed the *ESI* entrance thresholds in weaker research areas in which nations failed to collect a sufficient number of citations (Allik et al., 2020a,b). To correct these implausible rankings, we proposed to take also into account the number of research areas in which each country/territory failed to exceed the *ESI* entrance thresholds (Allik et al., 2020b). This was an improvement that, however, did not eliminate problematic rankings entirely. In this study, we proposed a novel approach according to which the scientific impact can be derived from the MDS analysis of (dis)similarities between the disciplinary profiles of the mean citation rate. The scientific impact was derived from a matrix of (dis)similarities between disciplinary profiles as a dimension of a recovered geometric space, which characterized the quality of sciences surprisingly adequately without artificially increasing the impact by withdrawing data in weaker research areas. Because shapes of the disciplinary profiles seemed to be irrelevant, only the cumulative citation rate across all disciplines matters in achieving a position in the science impact ranking.

There are several limitations in this study. The decision to include countries that were able publish 3,000 (instead of the previously used 4,000) or more papers during the 11-year period was a voluntary decision. However, some tests with a different number of countries demonstrated that the final plot of the MDS was invariant to this number and preserved its general configuration. Another potentially problematic decision was to replace unrepresented fields with the zero citation rates. We can only guess what the replacement zeros with the actual citation frequencies, which are expectedly close to nil anyway, would have resulted. Unfortunately, the *ESI* does not provide information about the number of publication and their citation rates that were left behind the entrance thresholds. Although we are among the first who noticed that the problem of spurious country rankings may be created by the *ESI*'s most precious property—focusing exclusively on the top of the citation distribution—we have very little information that the application of MDS to the disciplinary profiles provides the best answer to the problem. In one of our previous papers (Allik et al., 2020b), we already tried to correct rankings by taking into account in how many research areas each country/territory has failed to exceeded the entrance thresholds of the *ESI*. Although the spurious rankings were diminished, the improvement was less spectacular compared with the MDS of the disciplinary profiles used in this study. Additional studies are needed to establish what the best formula would be taking missing research fields into account.

## Data Availability Statement

All datasets generated for this study are included in the article.

## Author Contributions

All authors listed have made a substantial, direct and intellectual contribution to the work, and approved it for publication.

## Conflict of Interest

The authors declare that the research was conducted in the absence of any commercial or financial relationships that could be construed as a potential conflict of interest.
